# 2-Amino­anilinium picrate

**DOI:** 10.1107/S1600536810047057

**Published:** 2010-11-20

**Authors:** Rong Peng, Yanping Zhao

**Affiliations:** a Department of Chemistry and Biology, Xiangfan University, Xiangfan 441053, People’s Republic of China

## Abstract

In the title compound, C_6_H_9_N_2_^+^·C_6_H_2_N_3_O_7_^−^, the three nitro groups of the anion are twisted from the central benzene ring at dihedral angles of 5.4 (1), 27.1 (1) and 32.9 (1)°. In the crystal, inter­molecular N—H⋯O, N—H⋯(O,O) and N—H⋯N hydrogen bonds link the cations and anions into layers parallel to the *bc* plane.

## Related literature

For the crystal structures of picric acid complexes, see: Harrison *et al.* (2007 ▶); Li (2009 ▶); Saminathan *et al.* (2007 ▶); Sivaramkumar *et al.* (2010 ▶). For their conformational features and charge-transfer processes, see: Nagata *et al.* (1995 ▶); Smith *et al.* (2004 ▶).
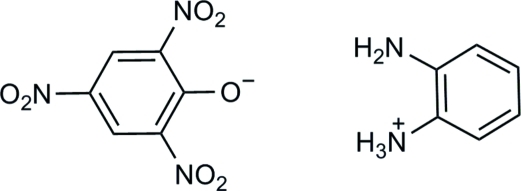


## Experimental

### 

#### Crystal data

C_6_H_9_N_2_^+^·C_6_H_2_N_3_O_7_^−^
*M* *_r_* = 337.26
Monoclinic, 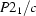

*a* = 13.2938 (11) Å
*b* = 6.9959 (6) Å
*c* = 15.2967 (13) Å
β = 92.629 (1)°
*V* = 1421.1 (2) Å^3^
*Z* = 4
Mo *K*α radiation
μ = 0.13 mm^−1^
*T* = 298 K
0.16 × 0.12 × 0.10 mm

#### Data collection

Bruker SMART APEX CCD area-detector diffractometer
Absorption correction: multi-scan (*SADABS*; Sheldrick, 1997 ▶) *T*_min_ = 0.979, *T*_max_ = 0.987
16897 measured reflections
3514 independent reflections
2394 reflections with *I* > 2σ(*I*)
*R*_int_ = 0.063

#### Refinement

*R*[*F* ^2^ > 2σ(*F* ^2^)] = 0.044
*wR*(*F* ^2^) = 0.120
*S* = 0.93
3514 reflections
232 parameters
H atoms treated by a mixture of independent and constrained refinement
Δρ_max_ = 0.22 e Å^−3^
Δρ_min_ = −0.29 e Å^−3^

### 

Data collection: *SMART* (Bruker, 2001 ▶); cell refinement: *SAINT* (Bruker, 1999 ▶); data reduction: *SAINT*; program(s) used to solve structure: *SHELXS97* (Sheldrick, 2008 ▶); program(s) used to refine structure: *SHELXL97* (Sheldrick, 2008 ▶); molecular graphics: *SHELXTL* (Sheldrick, 2008 ▶); software used to prepare material for publication: *SHELXTL*.

## Supplementary Material

Crystal structure: contains datablocks I, global. DOI: 10.1107/S1600536810047057/cv2788sup1.cif

Structure factors: contains datablocks I. DOI: 10.1107/S1600536810047057/cv2788Isup2.hkl

Additional supplementary materials:  crystallographic information; 3D view; checkCIF report

## Figures and Tables

**Table 1:** Hydrogen-bond geometry (Å, °)

*D*—H⋯*A*	*D*—H	H⋯*A*	*D*⋯*A*	*D*—H⋯*A*
N1—H1*A*⋯O1	0.922 (18)	2.048 (19)	2.9607 (18)	170.0 (14)
N2—H2*A*⋯O1	0.946 (16)	1.852 (17)	2.7731 (16)	163.9 (14)
N2—H2*A*⋯O7	0.946 (16)	2.514 (15)	2.8558 (17)	101.4 (11)
N1—H1*B*⋯O7^i^	0.813 (18)	2.424 (19)	3.2264 (17)	169.6 (16)
N2—H2*A*⋯O1^ii^	0.946 (16)	2.581 (16)	2.9872 (18)	106.2 (11)
N2—H2*B*⋯O2^ii^	0.858 (17)	2.448 (16)	3.122 (2)	135.9 (14)
N2—H2*B*⋯O6^iii^	0.858 (17)	2.556 (16)	2.9956 (17)	112.9 (12)
N2—H2*C*⋯N1^iv^	0.858 (18)	2.063 (18)	2.904 (2)	166.1 (16)

Symmetry codes: (i) 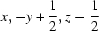; (ii) 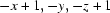; (iii) 
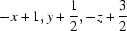
; (iv) 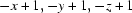.

**Fig. 1.**
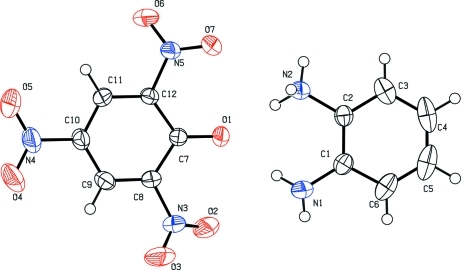
The molecular structure of (I) showing the atom-numbering scheme. Displacement ellipsoids are drawn at the 30% probability level.

## supplementary crystallographic information

### Comment

Picric acid is widely used in munitions and explosives. In microscopy, it also serves as a reagent for staining samples, *e.g.*, Gram staining. The crystal structures of a large number of picrate salts and picric acid complexes have been studied (Harrison *et al.*, 2007; Li, 2009; Saminathan *et al.*, 2007; Sivaramkumar *et al.*, 2010) to understand the conformational features and charge transfer processes (Nagata *et al.*, 1995; Smith *et al.*, 2004). We herein report the crystal structure of the title compound (I) (Fig. 1).

In (I), three nitro groups of the anion are twisted from the central benzene ring at 5.4 (1), 27.1 (1) and 32.9 (1)°, respectively. In the crystal structure, intermolecular N—H···O and N—H···N hydrogen bonds (Table 1) link cations and anions into layers parallel to *bc* plane.

### Experimental

Benzene-1,2-diamine (0.32 g, 3.0 mmol) and picric acid (0.69 g, 3.0 mmol) were mixed in 15 ml e thanol. The mixture was kept at room temperature for two weeks, after which time needle like yellow crystals (0.16 *x* 0.12 *x* 0.10 mm) suitable for single-crystal X-ray diffraction were obtained.

### Refinement

The O– and N-bound H atoms were located in a difference map and refined isotropically. The remaining H atoms were positioned geometrically (C—H = 0.95 Å) and allowed to ride on their parent atoms, with *U*_iso_(H) = 1.2 *U*_eq_(C).

### Crystal data

**Table:**

C_6_H_9_N_2_^+^·C_6_H_2_N_3_O_7_^−^	*F*(000) = 696
*M**_r_* = 337.26	*D*_x_ = 1.576 Mg m^−^^3^
Monoclinic, *P*2_1_/*c*	Mo *K*α radiation, λ = 0.71073 Å
Hall symbol: -P 2ybc	Cell parameters from 3836 reflections
*a* = 13.2938 (11) Å	θ = 2.7–24.9°
*b* = 6.9959 (6) Å	µ = 0.13 mm^−^^1^
*c* = 15.2967 (13) Å	*T* = 298 K
β = 92.629 (1)°	Block, yellow
*V* = 1421.1 (2) Å^3^	0.16 × 0.12 × 0.10 mm
*Z* = 4	

### Data collection

**Table:**

Bruker SMART APEX CCD area-detector diffractometer	3514 independent reflections
Radiation source: fine-focus sealed tube	2394 reflections with *I* > 2σ(*I*)
graphite	*R*_int_ = 0.063
phi and ω scans	θ_max_ = 28.3°, θ_min_ = 2.7°
Absorption correction: multi-scan (*SADABS*; Sheldrick, 1997)	*h* = −17→17
*T*_min_ = 0.979, *T*_max_ = 0.987	*k* = −9→9
16897 measured reflections	*l* = −20→20

### Refinement

**Table:**

Refinement on *F*^2^	Primary atom site location: structure-invariant direct methods
Least-squares matrix: full	Secondary atom site location: difference Fourier map
*R*[*F*^2^ > 2σ(*F*^2^)] = 0.044	Hydrogen site location: inferred from neighbouring sites
*wR*(*F*^2^) = 0.120	H atoms treated by a mixture of independent and constrained refinement
*S* = 0.93	*w* = 1/[σ^2^(*F*_o_^2^) + (0.0701*P*)^2^] where *P* = (*F*_o_^2^ + 2*F*_c_^2^)/3
3514 reflections	(Δ/σ)_max_ < 0.001
232 parameters	Δρ_max_ = 0.22 e Å^−^^3^
0 restraints	Δρ_min_ = −0.29 e Å^−^^3^

### Special details

**Table:**

Geometry. All e.s.d.'s (except the e.s.d. in the dihedral angle between two l.s. planes) are estimated using the full covariance matrix. The cell e.s.d.'s are taken into account individually in the estimation of e.s.d.'s in distances, angles and torsion angles; correlations between e.s.d.'s in cell parameters are only used when they are defined by crystal symmetry. An approximate (isotropic) treatment of cell e.s.d.'s is used for estimating e.s.d.'s involving l.s. planes.
Refinement. Refinement of *F*^2^ against ALL reflections. The weighted *R*-factor *wR* and goodness of fit *S* are based on *F*^2^, conventional *R*-factors *R* are based on *F*, with *F* set to zero for negative *F*^2^. The threshold expression of *F*^2^ > σ(*F*^2^) is used only for calculating *R*-factors(gt) *etc*. and is not relevant to the choice of reflections for refinement. *R*-factors based on *F*^2^ are statistically about twice as large as those based on *F*, and *R*- factors based on ALL data will be even larger.

### Fractional atomic coordinates and isotropic or equivalent isotropic displacement parameters (Å^2^)

**Table:**

	*x*	*y*	*z*	*U*_iso_*/*U*_eq_	
C1	0.38865 (11)	0.36646 (19)	0.40921 (9)	0.0336 (3)	
C2	0.34942 (10)	0.3253 (2)	0.48958 (9)	0.0325 (3)	
C3	0.24721 (11)	0.3359 (2)	0.50234 (12)	0.0467 (4)	
H3	0.2225	0.3084	0.5568	0.056*	
C4	0.18224 (13)	0.3879 (2)	0.43324 (16)	0.0612 (5)	
H4	0.1133	0.3938	0.4407	0.073*	
C5	0.22011 (15)	0.4306 (2)	0.35353 (15)	0.0617 (5)	
H5	0.1764	0.4655	0.3071	0.074*	
C6	0.32123 (14)	0.4227 (2)	0.34162 (11)	0.0484 (4)	
H6	0.3455	0.4553	0.2875	0.058*	
C7	0.68910 (10)	0.11086 (18)	0.55689 (8)	0.0280 (3)	
C8	0.77757 (10)	0.1384 (2)	0.50680 (9)	0.0315 (3)	
C9	0.87421 (11)	0.1403 (2)	0.54126 (9)	0.0361 (3)	
H9	0.9277	0.1672	0.5061	0.043*	
C10	0.89116 (10)	0.1016 (2)	0.62926 (9)	0.0370 (4)	
C11	0.81281 (10)	0.0667 (2)	0.68247 (9)	0.0346 (3)	
H11	0.8253	0.0365	0.7412	0.042*	
C12	0.71596 (10)	0.07703 (19)	0.64812 (8)	0.0291 (3)	
N1	0.49196 (10)	0.3622 (2)	0.39598 (9)	0.0406 (3)	
H1A	0.5270 (13)	0.274 (2)	0.4304 (11)	0.049*	
H1B	0.5052 (13)	0.351 (2)	0.3449 (12)	0.049*	
N2	0.41690 (10)	0.2695 (2)	0.56298 (8)	0.0367 (3)	
H2A	0.4726 (13)	0.195 (2)	0.5473 (10)	0.044*	
H2B	0.3848 (12)	0.218 (2)	0.6043 (11)	0.044*	
H2C	0.4430 (12)	0.373 (3)	0.5843 (11)	0.044*	
N3	0.76527 (10)	0.1700 (2)	0.41270 (8)	0.0436 (3)	
N4	0.99307 (10)	0.0980 (2)	0.66670 (10)	0.0588 (4)	
N5	0.63727 (9)	0.04886 (18)	0.70943 (8)	0.0365 (3)	
O1	0.60100 (7)	0.11173 (15)	0.52432 (6)	0.0377 (3)	
O2	0.69569 (9)	0.0908 (2)	0.37119 (7)	0.0630 (4)	
O3	0.82684 (10)	0.2717 (2)	0.37879 (8)	0.0740 (4)	
O4	1.06230 (9)	0.1404 (2)	0.61973 (10)	0.0814 (5)	
O5	1.00595 (10)	0.0516 (3)	0.74296 (10)	0.1001 (6)	
O6	0.65667 (9)	−0.04467 (18)	0.77549 (7)	0.0549 (3)	
O7	0.55494 (8)	0.1226 (2)	0.69490 (7)	0.0632 (4)	

### Atomic displacement parameters (Å^2^)

**Table:**

	*U*^11^	*U*^22^	*U*^33^	*U*^12^	*U*^13^	*U*^23^
C1	0.0363 (8)	0.0312 (8)	0.0327 (7)	−0.0020 (6)	−0.0049 (6)	−0.0030 (6)
C2	0.0295 (7)	0.0315 (7)	0.0361 (8)	−0.0009 (6)	−0.0016 (6)	−0.0024 (6)
C3	0.0334 (8)	0.0406 (9)	0.0666 (11)	0.0004 (7)	0.0061 (8)	−0.0058 (8)
C4	0.0295 (9)	0.0461 (10)	0.1064 (17)	0.0022 (7)	−0.0120 (10)	−0.0108 (11)
C5	0.0573 (12)	0.0437 (10)	0.0804 (14)	0.0053 (8)	−0.0370 (11)	−0.0043 (9)
C6	0.0590 (11)	0.0405 (9)	0.0436 (9)	−0.0007 (8)	−0.0204 (8)	0.0008 (7)
C7	0.0277 (7)	0.0304 (7)	0.0260 (7)	0.0019 (5)	0.0014 (5)	−0.0020 (5)
C8	0.0340 (7)	0.0363 (8)	0.0244 (7)	0.0001 (6)	0.0034 (6)	0.0000 (6)
C9	0.0303 (8)	0.0436 (9)	0.0352 (8)	−0.0035 (6)	0.0088 (6)	−0.0026 (6)
C10	0.0254 (7)	0.0499 (9)	0.0355 (8)	−0.0007 (6)	−0.0014 (6)	−0.0066 (7)
C11	0.0335 (8)	0.0458 (9)	0.0243 (7)	0.0019 (6)	−0.0010 (6)	−0.0029 (6)
C12	0.0262 (7)	0.0372 (8)	0.0242 (7)	−0.0009 (6)	0.0043 (5)	−0.0023 (5)
N1	0.0413 (8)	0.0535 (8)	0.0273 (6)	−0.0016 (6)	0.0048 (6)	0.0025 (6)
N2	0.0340 (7)	0.0485 (8)	0.0280 (6)	0.0019 (6)	0.0048 (5)	−0.0027 (6)
N3	0.0453 (8)	0.0586 (8)	0.0276 (6)	0.0047 (7)	0.0078 (6)	0.0068 (6)
N4	0.0280 (7)	0.0973 (12)	0.0506 (9)	0.0024 (7)	−0.0036 (7)	−0.0095 (8)
N5	0.0316 (7)	0.0518 (8)	0.0263 (6)	−0.0020 (6)	0.0043 (5)	−0.0022 (5)
O1	0.0271 (5)	0.0558 (7)	0.0299 (5)	0.0053 (4)	−0.0026 (4)	−0.0021 (5)
O2	0.0590 (8)	0.1025 (11)	0.0270 (6)	−0.0075 (7)	−0.0025 (6)	−0.0022 (6)
O3	0.0754 (9)	0.1017 (11)	0.0460 (7)	−0.0163 (8)	0.0145 (7)	0.0306 (7)
O4	0.0284 (7)	0.1396 (14)	0.0766 (10)	−0.0101 (7)	0.0079 (6)	−0.0095 (9)
O5	0.0424 (8)	0.199 (2)	0.0570 (9)	0.0039 (10)	−0.0191 (6)	0.0133 (11)
O6	0.0575 (8)	0.0729 (8)	0.0355 (6)	0.0063 (6)	0.0145 (5)	0.0171 (6)
O7	0.0332 (6)	0.1180 (11)	0.0390 (7)	0.0164 (7)	0.0102 (5)	0.0097 (7)

### Geometric parameters (Å, °)

**Table:**

C1—C2	1.387 (2)	C9—H9	0.9300
C1—C6	1.393 (2)	C10—C11	1.373 (2)
C1—N1	1.3977 (19)	C10—N4	1.447 (2)
C2—C3	1.383 (2)	C11—C12	1.3699 (19)
C2—N2	1.4579 (19)	C11—H11	0.9300
C3—C4	1.383 (3)	C12—N5	1.4501 (17)
C3—H3	0.9300	N1—H1A	0.922 (18)
C4—C5	1.373 (3)	N1—H1B	0.813 (18)
C4—H4	0.9300	N2—H2A	0.946 (16)
C5—C6	1.366 (3)	N2—H2B	0.858 (17)
C5—H5	0.9300	N2—H2C	0.858 (18)
C6—H6	0.9300	N3—O3	1.2182 (17)
C7—O1	1.2517 (15)	N3—O2	1.2295 (17)
C7—C12	1.4441 (18)	N4—O5	1.215 (2)
C7—C8	1.4457 (19)	N4—O4	1.2296 (19)
C8—C9	1.366 (2)	N5—O7	1.2211 (16)
C8—N3	1.4580 (18)	N5—O6	1.2214 (15)
C9—C10	1.381 (2)		
			
C2—C1—C6	117.44 (14)	C11—C10—C9	121.21 (13)
C2—C1—N1	122.40 (13)	C11—C10—N4	118.98 (13)
C6—C1—N1	120.08 (15)	C9—C10—N4	119.81 (13)
C3—C2—C1	121.70 (14)	C12—C11—C10	119.25 (13)
C3—C2—N2	118.64 (14)	C12—C11—H11	120.4
C1—C2—N2	119.66 (12)	C10—C11—H11	120.4
C4—C3—C2	119.30 (17)	C11—C12—C7	124.44 (12)
C4—C3—H3	120.4	C11—C12—N5	115.98 (12)
C2—C3—H3	120.4	C7—C12—N5	119.57 (12)
C5—C4—C3	119.63 (17)	C1—N1—H1A	113.9 (10)
C5—C4—H4	120.2	C1—N1—H1B	113.6 (12)
C3—C4—H4	120.2	H1A—N1—H1B	111.0 (16)
C6—C5—C4	120.84 (17)	C2—N2—H2A	114.5 (10)
C6—C5—H5	119.6	C2—N2—H2B	111.7 (11)
C4—C5—H5	119.6	H2A—N2—H2B	112.2 (15)
C5—C6—C1	121.06 (17)	C2—N2—H2C	107.0 (11)
C5—C6—H6	119.5	H2A—N2—H2C	104.6 (15)
C1—C6—H6	119.5	H2B—N2—H2C	106.2 (16)
O1—C7—C12	124.81 (12)	O3—N3—O2	123.18 (13)
O1—C7—C8	123.88 (12)	O3—N3—C8	117.51 (14)
C12—C7—C8	111.28 (12)	O2—N3—C8	119.29 (13)
C9—C8—C7	124.77 (13)	O5—N4—O4	123.30 (15)
C9—C8—N3	116.13 (13)	O5—N4—C10	118.21 (15)
C7—C8—N3	119.10 (12)	O4—N4—C10	118.49 (15)
C8—C9—C10	118.86 (13)	O7—N5—O6	122.04 (12)
C8—C9—H9	120.6	O7—N5—C12	119.47 (12)
C10—C9—H9	120.6	O6—N5—C12	118.46 (12)
			
C6—C1—C2—C3	1.1 (2)	N4—C10—C11—C12	177.54 (14)
N1—C1—C2—C3	177.94 (14)	C10—C11—C12—C7	4.1 (2)
C6—C1—C2—N2	−178.80 (13)	C10—C11—C12—N5	−176.71 (13)
N1—C1—C2—N2	−2.0 (2)	O1—C7—C12—C11	176.54 (14)
C1—C2—C3—C4	0.3 (2)	C8—C7—C12—C11	−1.78 (19)
N2—C2—C3—C4	−179.72 (14)	O1—C7—C12—N5	−2.6 (2)
C2—C3—C4—C5	−0.9 (2)	C8—C7—C12—N5	179.07 (12)
C3—C4—C5—C6	−0.1 (3)	C9—C8—N3—O3	−30.9 (2)
C4—C5—C6—C1	1.6 (3)	C7—C8—N3—O3	148.08 (14)
C2—C1—C6—C5	−2.1 (2)	C9—C8—N3—O2	147.45 (14)
N1—C1—C6—C5	−178.99 (14)	C7—C8—N3—O2	−33.5 (2)
O1—C7—C8—C9	179.16 (14)	C11—C10—N4—O5	4.8 (3)
C12—C7—C8—C9	−2.51 (19)	C9—C10—N4—O5	−175.38 (17)
O1—C7—C8—N3	0.2 (2)	C11—C10—N4—O4	−175.78 (16)
C12—C7—C8—N3	178.56 (12)	C9—C10—N4—O4	4.0 (2)
C7—C8—C9—C10	4.3 (2)	C11—C12—N5—O7	152.66 (14)
N3—C8—C9—C10	−176.74 (13)	C7—C12—N5—O7	−28.1 (2)
C8—C9—C10—C11	−1.8 (2)	C11—C12—N5—O6	−25.50 (19)
C8—C9—C10—N4	178.44 (14)	C7—C12—N5—O6	153.72 (13)
C9—C10—C11—C12	−2.2 (2)		

### Hydrogen-bond geometry (Å, °)

**Table:**

*D*—H···*A*	*D*—H	H···*A*	*D*···*A*	*D*—H···*A*
N1—H1A···O1	0.922 (18)	2.048 (19)	2.9607 (18)	170.0 (14)
N2—H2A···O1	0.946 (16)	1.852 (17)	2.7731 (16)	163.9 (14)
N2—H2A···O7	0.946 (16)	2.514 (15)	2.8558 (17)	101.4 (11)
N1—H1B···O7^i^	0.813 (18)	2.424 (19)	3.2264 (17)	169.6 (16)
N2—H2A···O1^ii^	0.946 (16)	2.581 (16)	2.9872 (18)	106.2 (11)
N2—H2B···O2^ii^	0.858 (17)	2.448 (16)	3.122 (2)	135.9 (14)
N2—H2B···O6^iii^	0.858 (17)	2.556 (16)	2.9956 (17)	112.9 (12)
N2—H2C···N1^iv^	0.858 (18)	2.063 (18)	2.904 (2)	166.1 (16)

Symmetry codes: (i) *x*, −*y*+1/2, *z*−1/2; (ii) −*x*+1, −*y*, −*z*+1; (iii) −*x*+1, *y*+1/2, −*z*+3/2; (iv) −*x*+1, −*y*+1, −*z*+1.
